# Back to the roots: protocol for the photoautotrophic micropropagation of medicinal *Cannabis*

**DOI:** 10.1007/s11240-019-01635-1

**Published:** 2019-06-05

**Authors:** Andrea Kodym, Christian J. Leeb

**Affiliations:** 1 Department of Pharmacognosy, University of Vienna, 1090 Vienna, Austria; 2 Core Facility Botanical Garden, University of Vienna, 1030 Vienna, Austria

**Keywords:** Aseptic culture, In vitro, *Cannabis sativa*, Plant tissue culture, Vegetative propagation

## Abstract

The aim of this protocol was to develop an alternative in vitro propagation system for *Cannabis sativa* L. by mimicking nursery-based vegetative propagation. Photoautotrophic micropropagation (PAM) was achieved on rockwool blocks as substrate combined with commercially available fertilizer suitable for cannabis cultivation. Stock plants were initiated after sterilisation in forced-ventilated glass jars which then provided a continuous supply of shoot tip and nodal cuttings. A 97.5% rooting rate of in vitro shoot tip cuttings and successful acclimatisation were achieved within 3 weeks in glass vessels with passive ventilation.

## Key message

Cannabis is a difficult plant to grow in vitro, in particular habit is strongly influenced by culture conditions. A photo-autotropic micropropagation system was developed to overcome culture-induced problems.

## Introduction

*Cannabis sativa* L., rightfully also labelled as ‘the plant of the thousand and one molecules’ (Andre et al. 2016) has had a long tradition as a source of fibre, food and as a recreational and medicinal drug in the history of mankind. As more clinical studies prove its pharmaceutical value, governments are legalising medicinal cannabis and as a consequence demand is rising steadily (INCB, 2018).

Micropropagation as an aseptic technique has a high potential in cannabis propagation. It allows the rapid propagation of uniform materials of elite chemotypes free of diseases and pests. Valuable mother stock plants can be safe-guarded in culture and they enable shipment and transfer of high-quality germplasm. Although several micropropagation protocols have been published (Casano and Grassi 2009; Wang et al. 2009; Lata et al. 2016a, b), in vitro propagation of cannabis is challenging (Lata et al. 2017) and can result in off-types whose morphology does not resemble normal plantlets. The aim of this study was to find a reliable and productive micropropagation system that produces vigorous plants of high quality.

## Methods

### Initiation of aseptic stock cultures

1. Raise disease and pest-free cannabis stock plants in a glass house between 18 and 30 °C under a minimum of 18 h of light.
2. Take shoot tip cuttings below the third node (80–100 mm in size), remove the lowest leaf and rinse in tap water.
3. Surface sterilise in 0.5% NaOCl for 20 min in the laminar hood.
4. Rinse the plant material with sterile water and wash another three times for 10 min.
5. Prepare explants consisting of a shoot tip and two nodes (30–40 mm in size). Trim back leaf blades by two-thirds.
6. Transfer to either sterile forced ventilated glass jars or RITA containers (Vitropic, CIRAD, France).
7. Place the cultures in the growth chamber at 25 ± 1 °C with a 16 h photoperiod. Light intensity (PPFD—photosynthetic photon flux density) inside the vessels was around 70 µmol m^−2^ s^−1^.

### Maintenance of stock plants in forced ventilated glass jars

1. Place one quarter of a rockwool block (Grodan, The Netherlands, size 100 × 100 × 650 mm) into a glass preservation jar (1.5 or 2 L, 220 or 240 mm height) with a hinged lock and a rubber seal (Le Parfait super jars, France). The lid of the jar was modified so it could be forced-ventilated through sterile filters (Fig. 1a).

**Fig. 1:**
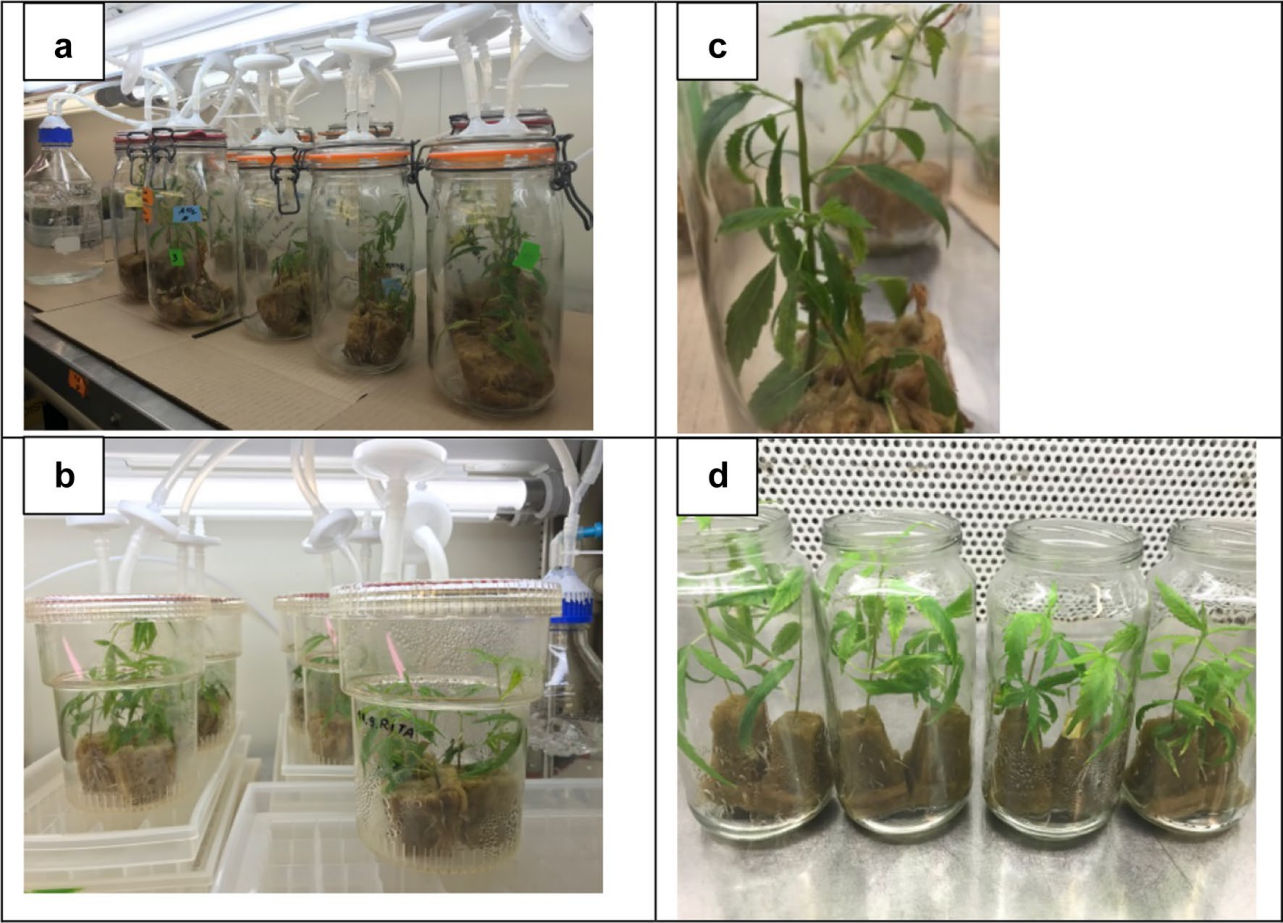
**a** Forced ventilated glass jars, **b** RITA system after 3 weeks culture, **c** regrowth of pruned shoots, **d** rooted shoots at time of transfer to glass house
2. Wet block with 20 mL of distilled water.
3. Autoclave the jars at 121 °C for 15 min.
4. After autoclaving, wet the block with 160 mL of sterile distilled water and plant three shoot tip cuttings into each block under sterile conditions.
5. Connect the closed jars via tubing to an air pump at a pressure of 1 bar. The air flow needs to pass by a bottle with distilled water to provide moisturised air. (Figure 1a) let the pump operate for 14 h per day, starting 2 h after the onset of the light period.
6. After 2 weeks, add 20 mL of nutrient solution (Canna Aqua Vega Fertilizer A + B Set, The Netherlands) to each jar; the nutrient solution is prepared with distilled water according to the manufacture’s instruction and the pH is set to 6.0–6.2 with NaOH before autoclaving at 121 °C for 15 min. The EC (electrical conductivity) of the nutrient solution is around 1.2.
7. After 6 to 7 weeks, tip-prune the plantlets for the first time and remove any wilted leaves.
8. Repeatedly prune plantlets as they grow bigger and use cuttings for rooting or setting up more stock plants.
9. Add water or nutrient solution (alternating) when rockwool blocks start to try out.

### Maintenance of stock plants using the RITA-system

1. Set up a RITA container without the basket and bell with three rockwool blocks each (size 36 × 36 x 36 mm).
2. Wet blocks with 15 mL of distilled water.
3. Autoclave the containers at 121 °C for 15 min.
4. After autoclaving add 75 mL of sterile nutrient solution (described above) and plant two shoot tip cuttings into each block (6 explants per container) under sterile conditions.
5. Connect the jars via tubing to an air pump at a pressure of 1 bar. The air flow needs to pass by a bottle with distilled water to provide moisturized air. (Figure 1b) let the pump operate for 14 h per day, starting 2 h after the onset of the light period.
6. After 3 weeks, tip-prune the plantlets for the first time and remove any wilted leaves.
7. Repeatedly prune plantlets as they grow bigger and use cuttings for rooting or setting up more stock plants.
8. Add water or nutrient solution (alternating) when rockwool blocks start to try out.

### Rooting and acclimatisation

1. Prepare 250 mL glass vessels (Hipp, Gmunden, Austria, 120 mm height) with vented lids (Magenta B-cap with 10 mm opening covered with adhesive microfiltration discs from TQPL, Hampshire, UK) with two rockwool blocks (size 20 x 20 x 40 mm).
2. Place the blocks upside down for stability and wet blocks with 10 mL of distilled water.
3. Autoclave vessels at 121 °C for 15 min.
4. After autoclaving, add 20 mL nutrient solution (described above).
5. Harvest shoot tips with three to four nodes from the in vitro stock plants and insert them into rockwool. Trim back large leaves. Use one shoot tip per block.
6. Place the cultures back in the growth chamber.
7. After 2 weeks add 10 mL of water to each jar.
8. Place lids upside down for the next 3 days to initiate the acclimatisation process.
9. Remove the lids completely and move the jars to a shelf with low lighting for the next 2 days to avoid drying out.
10. Pot up plantlets with the block into a general peat based potting mix into 100 mm pots (360 mL) and place them in a glass house with hand watering and a photoperiod of 16 h.

## Results and discussion

After 5 weeks culture in forced-ventilated glass jars, 95% of the newly initiated shoot tips had grown on and were rooted and well developed. Cuttings from the in vitro stock plants were best taken once three or more nodes had regrown so that the lowest node(s) with axillary buds could remain for re-sprouting and to manage plant height. The stock cultures could be maintained this way for at least 6 months. The self-built preservation jars were more suited for the culture of cannabis as they provided more head space, on the other hand the RITA system was more practicable in terms of handling because of the wide opening. Shoot tip cuttings were the preferred source over nodal cuttings for the same reasons as in standard nursery propagation (Table 1). The biggest improvement through this PAM system was on plant quality.

**Table 1:** Troubleshooting for photoautotrophic micropropagation of cannabis

Problem	Possible reason	Solution
In vitro plants are infected	Pests were carried over from donor plants	Treat donor plants in glasshouse with pesticides
	Sterilisation was not sufficient	Increase concentration of NaOCl solution or time span for sterilisation
Plants are starting to wilt	Blocks are too dry Humidity inside vessel is too low	Add water or nutrient solution to the vessel Decrease air pressure
Plants are deteriorating	Blocks are too wet	Don’t have access liquid stand in the vessels
Leaves are turning yellow or plants are stunted	Nutrient deficiency	Add nutrient solution

## Conclusions

- A photoautotrophic in vitro propagation system has been developed that simply relies on industry-based fertilizer, rockwool blocks as substrate and forced ventilation.
- Stock plants were initiated only once and provided a steady supply of shoot tips for propagation for at least 6 months.
- In vitro plantlets were of excellent quality: leaves were dark green and compound, shoots had clearly defined internodes and there were no signs of hyperhydricity. Plantlets resembled their nursery analogues (Fig. 1c).
- 97.5% of in vitro shoot tip cuttings were rooted and acclimatized within 3 weeks inside the growth chamber.
- Survival of rooted cuttings in the glass house was 100%.
- The whole process did not require any sugars or vitamins. Not having to include sugars has two big advantages, (1) cultures are less prone to microbial contamination and (2) the acclimatisation step can be significantly reduced (Nguyen et al. 2016).
- The whole process did not require any plant growth regulators either which almost certainly preserves genetic stability and overcomes fears of users that spontaneous mutations or somaclonal variation occur which are more likely associated with the use of growth regulators (Pierik 1997).
